# Evaluation of the Rheological Behavior and the Development of Performance Equations of Asphalt Composites Produced with Titanium Dioxide and Zinc Oxide Nanoparticles

**DOI:** 10.3390/nano13020288

**Published:** 2023-01-10

**Authors:** João Victor Staub de Melo, Alexandre Luiz Manfro, Breno Salgado Barra, Natália Dell’Antonio Cadorin, Wellington Borba Broering

**Affiliations:** Department of Civil Engineering, Federal University of Santa Catarina, Rua João Pio Duarte Silva, Florianópolis 88040-970, SC, Brazil

**Keywords:** nanoparticles, titanium dioxide, zinc oxide, asphalt composites, rheological behavior, performance model, mathematical equations

## Abstract

This research evaluated the rheological behavior of conventional asphalt binders modified with TiO_2_ and ZnO nanoparticles and proposed mathematical equations for performance prediction. First, composites were evaluated at high temperatures to investigate the Performance Grade (PG), non-recoverable creep compliance, and Aging Index (AI). Subsequently, the fatigue damage tolerance was determined at a temperature of 20 °C through the Linear Amplitude Sweep (LAS) test. At high temperatures, for both nanoparticles, stiffness gain was observed as the nanomaterial content increased, evidenced by the increase in the dynamic shear modulus. This resulted in an increase in the Performance Grade and reduction in non-recoverable creep compliance, leading to greater resistance to permanent deformations. Furthermore, it was found that nanoparticles were able to reduce the effects of oxidation of the asphalt matrix, corroborated by the reduction of the Aging Index (AI). Regarding the fatigue damage tolerance, for both nanoparticles, an increase in performance was observed at low deformation amplitudes and a decrease at high deformation amplitudes. Finally, the analysis of each rheological parameter allowed to define the mathematical equations capable of predicting the performance of conventional asphalt binders when modified with nano-TiO_2_ or nano-ZnO.

## 1. Introduction

In the last decade, researchers began to study the incorporation of nanometric semiconductors in asphalt mixtures [1,2,3,4,5,6,7,8]. In this context, TiO_2_ and ZnO nanoparticles are among the most investigated semiconductors due to their low toxicity, high chemical stability, and adequate potential for oxidation processes [5].

One of the main methods for adding nanometric semiconductors to asphalt concrete consists of mixing nanoparticles with the asphalt binder [8]. In this process, the asphalt binder is heated until the desired viscosity is reached so that nanoparticles can be properly dispersed [9,10,11].

However, one must avoid that the properties of conventional materials are damaged due to modification. Thus, when adding new functionalities to the asphalt matrix, one must necessarily avoid compromising its mechanical performance, as it is necessary to investigate the mechanical and rheological parameters related to the developed composite [12].

The literature reports the impacts of adding TiO_2_ and ZnO nanoparticles to asphalt binders: nano-TiO_2_ decreases the penetration grade and increases the softening point of binders [13], increases toughness [14], increases the rutting resistance [15], increases stiffness [4], and improves resistance to aging of asphalt binders [16]; nano-ZnO increases the softening point and the binder viscosity [17], decreases the penetration grade [3], increases the anti-aging ability [18,19], improves the shear resistance [20], and the dispersion of copolymer SBS in the asphalt [21] and decreases the acidity of the asphalt binder [22].

Despite the existence of studies addressing the use of nanometric semiconductors as modifiers of asphalt matrices [9,23,24,25], there is still a lack of studies that comparatively analyze the mechanical/rheological performance of composites produced with nano-TiO_2_ and nano-ZnO. In this way, the definition of mathematical equations for predicting the performance of these composites can contribute to the area of knowledge, allowing the comparison between asphalt binders modified by TiO_2_ and ZnO nanoparticles.

Therefore, the present study evaluates the rheological behavior of asphaltic composites in order to propose mathematical equations for performance prediction. For this purpose, asphalt composites were produced from a conventional asphalt binder modified with nano-TiO_2_ and nano-ZnO, being evaluated for their mechanical performance in terms of susceptibility to permanent deformation and fatigue damage tolerance.

## 2. Materials and Methods

### 2.1. Materials

To carry out this study, the following materials were used: conventional asphalt binder obtained from “Presidente Getúlio Vargas” Refinery (REPAR/Petrobras, Araucária - PR, Brazil), zinc oxide (nano-ZnO), and titanium dioxide (nano-TiO_2_) (Nanostructured & Amorphous Material, Inc., Houston, TX, USA). Table 1 presents the asphalt binder properties.

**Figure 1:**
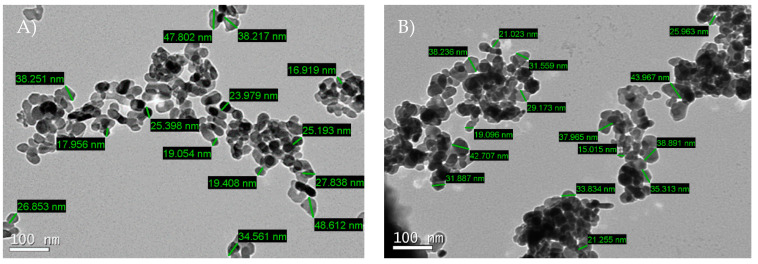
Nano-TiO_2_ (**A**) and nano-ZnO (**B**) micrographs obtained by transmission electron microscopy technique.

Nanoparticles were obtained from Nanostructured & Amorphous Material, Inc. (Houston, USA) and have the following characteristics:

- nano-TiO_2_: predominantly consisting of titanium and oxygen, also containing copper ≤ 3 ppmv, cadmium ≤ 8 ppmv, manganese ≤ 5 ppmv, lead ≤ 9 ppmv and arsenic ≤ 5 ppmv (Energy-Dispersive X-ray Spectroscopy); purity of 99%; ellipsoidal and spherical shape (Figure 1A); average size of individual particles of 29.3 ± 10.6 nm; specific surface area of 60 m^2^/g; density of 3.9 (20 °C); and pH between 5.5 and 5.7.
- nano-ZnO: predominantly consisting of zinc and oxygen, also containing copper ≤ 3 ppmv; cadmium ≤ 8 ppmv; manganese ≤ 5 ppmv; lead ≤ 9 ppmv and arsenic ≤ 5 ppmv (Energy-Dispersive X-ray Spectroscopy); purity of 99%; spherical shape (Figure 1B); average size of individual particles of 31.3 ± 8.7 nm; specific surface area of 40 m^2^/g; density of 5.6 (20 °C); and pH between 6.5 and 7.5.

**Figure 2:**
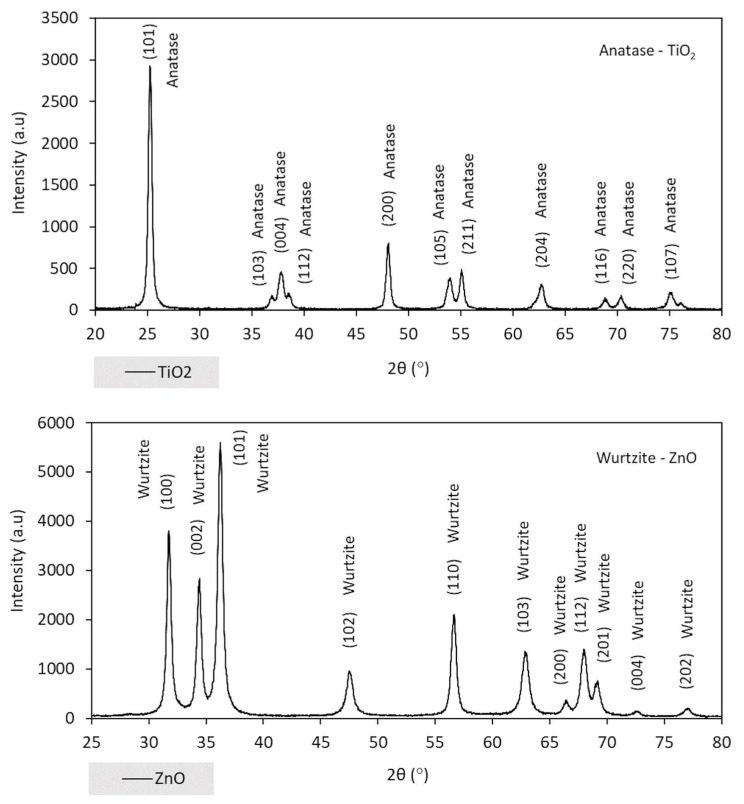
X-ray diffractograms: nano-TiO_2_ and nano-ZnO.

Nanoparticles were also characterized in terms of crystalline structure using the X-ray diffraction technique. According to diffractograms (Figure 2), 11 well-defined peaks are verified for both materials, indicating that TiO_2_ presents a tetragonal crystalline system (anatase) (according to the JCPDS 21-1272 catalog card [33,34,35,36,37]) and nano-ZnO presents a hexagonal crystalline system (wurtzite) (according to the JCPDS 79-2205 catalog card [38,39]).

**Figure 3:**
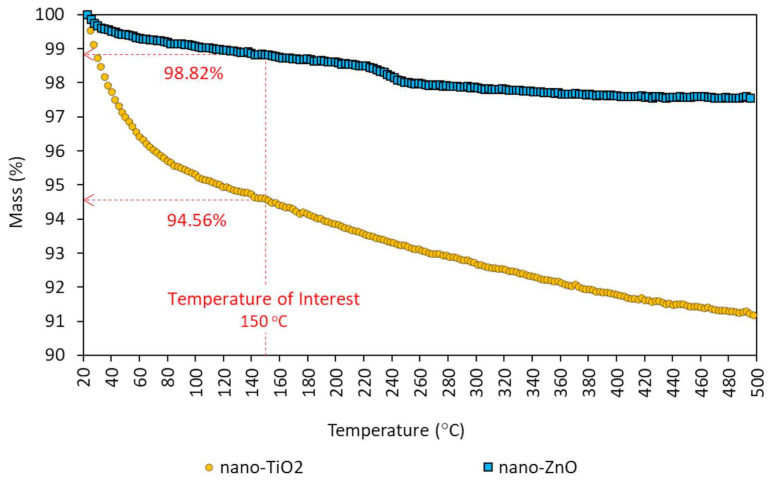
Thermogravimetric curve in nitrogen atmosphere: nano-TiO_2_ and nano-ZnO.

In addition to primary materials, five asphalt composites with the incorporation of nano-TiO_2_ (3%, 6%, 9%, 12% and 15%) and five with the incorporation of nano-ZnO (3%, 6%, 9%, 12% and 15%), in relation to the binder mass, were produced for this study. Nanomodification was carried out with an amount of binder of 300 mL at temperature of 150 ± 5 °C (apparent viscosity of 0.178 Pa.s), with high-shear mixer at a speed of 6000 rpm for 15 min. The modification temperature was defined as a function of the base binder viscosity, taking as reference the viscosity range used for the production of asphalt mixtures (0.150 to 0.190 Pa.s) [40]. The validation of the nanomodification temperature was performed through the thermogravimetry test shown in Figure 3, where the thermal stability of nanoparticles at the defined temperature was verified. According to Figure 3, the results showed that at 150 °C, there is mass loss of 5.44% (94.56%) and 1.18% (98.82%) for nano-TiO_2_ and nano-ZnO, respectively, caused only by the loss of structural and adsorbed water (sample dehydration), and that the degradation of materials occurs at temperatures above 500 °C.

### 2.2. Methods

To achieve the main aim of this research, the following methodological steps were established: (i) evaluation of the influence of nanoparticles on rheological parameters indicative of resistance to permanent deformation of the asphalt matrix; and (ii) performance of composites in terms of fatigue damage tolerance.

#### 2.2.1. Evaluation of the Influence of Nanoparticles on Rheological Parameters Indicative of Resistance to Permanent Deformation of the Asphalt Matrix

The effects of incorporating nanoparticles on the rheological parameters of the asphalt binder related to permanent deformation were evaluated using a dynamic shear rheometer (HR-2, TA Instruments, Discovery Hybrid Rheometer, New Castle, USA). For this, asphalt binder samples nanomodified with nano-TiO_2_ and nano-ZnO, as well as conventional binder (reference), were tested at high temperatures (58 °C, 64 °C, 70 °C, 76 °C and 82 °C). In this temperature range, the dynamic shear modulus (|G*|) and phase angle (delta—δ) were investigated in short-term aged samples (Rolling Thin-Film Oven Test—RTFOT [41]). Tests were conducted at a frequency of 10 rad/s (1.59 Hz), under controlled deformation of 10%, according to requirements of ASTM D6373 [26], ASTM D7643 [42], and ASTM D7175 standards [43]. Additionally, the performance of aged matrices (RTFOT [41]) was verified in the Multiple Stress Creep and Recovery (MSCR) test, according to ASTM D7405 [44], at temperatures of 58 °C and 64 °C; this temperature range is used to evaluate the resistance to permanent deformation of asphalt mixtures [45,46,47]. The parameters evaluated in this test were the recovery rate (%R_3.2_) and the non-recoverable creep compliance (Jnr_3.2_) at a stress of 3.2 kPa. For all tests carried out at high temperatures in the rheometer, samples with a geometry of 25 mm in diameter and gap of 1 mm in height were used in duplicates. In addition to this test, the effect of nanoparticles on the binder Aging Index (AI) was determined, obtained by the ratio between the rheological parameter |G*|/sin δ after and before (virgin condition) short-term aging in RTFOT.

Finally, based on laboratory results, equations were obtained to predict the behavior of the conventional binder as a function of the percentage of nanoparticle incorporation (TiO_2_ and ZnO) to the matrix. The following equations were developed:

- An equation for TiO_2_ and another for ZnO for predicting parameter |G*|/sin δ of the conventional aged binder (RTFOT) as a function of temperature and percentage of nano incorporation. The equations allow the classifying of the conventional binder with TiO_2_ or ZnO nanoparticles in relation to the High Temperature Performance Grade (PGH).
- An equation for TiO_2_ and another for ZnO for predicting the non-recoverable creep compliance (Jnr_3,2_) of the conventional aged binder (RTFOT) as a function of temperature and percentage of nano incorporation. The equations allow the classifying of the conventional binder with TiO_2_ or ZnO nanoparticles in relation to the traffic level, proposed by AASHTO M 332 [48].
- An equation for TiO_2_ and another for ZnO for predicting the influence of nanoparticles on the conventional binder Aging Index (AI) as a function of temperature and percentage of nano incorporation.

It should be noted that the equations are suitable for any asphalt matrix that has the following initial characteristics: penetration between 50 and 70 tenths of millimeter and/or PG 58-XX.

#### 2.2.2. Performance of Composites Regarding Fatigue Damage Tolerance

The effect of the asphalt binder modified with nano-TiO_2_ and nano-ZnO on the fatigue damage tolerance was determined using the Linear Amplitude Sweep (LAS) test (AASHTO TP 101 [49]) in aged residues (RTFOT [41]). Tests were conducted using the Discovery Hybrid Rheometer (HR-2, TA Instruments, New Castle, USA) at temperature of 20 °C, frequency of 10 Hz, and strain range from 0% to 30% (3100 loading cycles). The rupture criterion was defined as a 35% reduction in the |G*|·sin δ value of the intact material, as recommended by Chen, Zhang, and Bahia [50] and Chen and Bahia [51], thus obtaining the fatigue law (Nf = A(γ_max_)^B^) of asphalt matrices (reference and nanomodified). Samples were tested in duplicate, with a geometry of 8 mm in diameter and gap of 2 mm in height.

Finally, based on results, an equation for TiO_2_ and another for ZnO was obtained to predict the number of cycles to fatigue failure (Nf) of the conventional aged binder (RTFOT) as a function of the percentage of nano incorporation and deformation amplitude (γ_max_) (at temperature of 20 °C and frequency of 10 Hz). The equations allow for the verification of the behavior of the matrix in terms of damage tolerance for any asphalt matrix with the following initial characteristics: penetration between 50 and 70 tenths of millimeter and/or PG 58-XX.

## 3. Results and Discussion

### 3.1. Evaluation of the Influence of Nanoparticles on Rheological Parameters Indicative of Resistance to Permanent Deformation of the Asphalt Matrix

**Figure 4:**
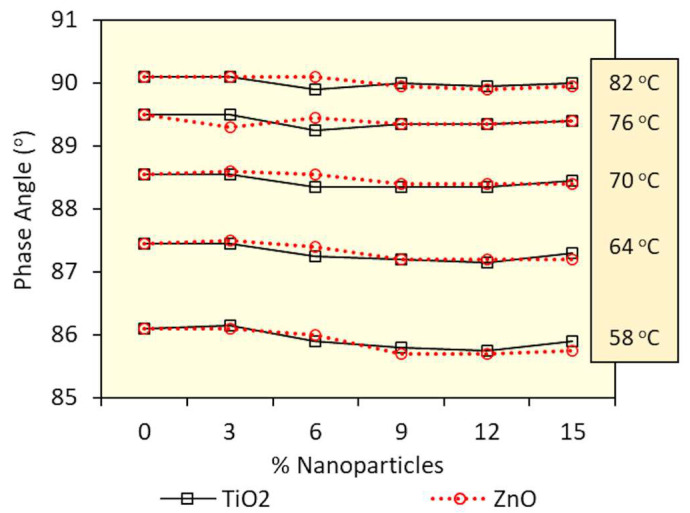
Comparison of the effect of the percentage of TiO_2_ and ZnO on the phase angle (δ) of the conventional binder short-term aged at high temperatures (58 °C to 82 °C).

**Figure 5:**
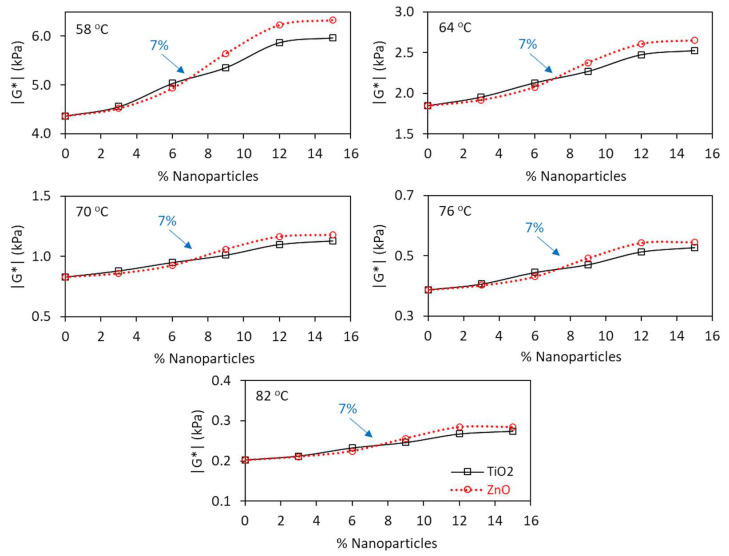
Comparison of the effect of the percentage of TiO_2_ and ZnO on the dynamic shear modulus (|G*|) of conventional binder short-term aged at high temperatures (58 °C to 82 °C).

Figure 4 and Figure 5 present, respectively, the phase angle and dynamic shear modulus results of nanomodified and reference asphalt matrices. Values are characteristic of short-term aged residues, investigated at high temperatures, between 58 °C and 82 °C.

Based on the results presented in Figure 4, it is possible to verify that none of the nanoparticles under study (TiO_2_ or ZnO) in the incorporation range of up to 15% provided significant changes in the phase angle of the original matrix. In addition, at all evaluated temperatures (58 °C to 82 °C), no tendency to increase or decrease was observed when nanoparticles were added, as well as no difference in behavior was observed regarding the type of nanoparticle used. These results allow us to conclude that nanoparticles, when added up to 15%, do not impact the elastic response of the matrix at high temperatures.

Regarding the dynamic shear modulus, it was observed in Figure 5 that the addition of nanoparticles increases the matrix stiffness at all temperatures; however, with greater impact at 58 °C and 64 °C.

The incorporation of nano-TiO_2_ or nano-ZnO in the asphalt matrix results in increase in the modulus due to the nature of nanoparticles, which are not viscous but crystalline, with a small dimension and high specific surface area, which facilitates the matrix reinforcement, increasing its consistency and, consequently, its stiffness. The phase angle does not change because nanoparticles cannot add elasticity to the matrix, since they are not elastic particles but crystalline solids. However, these nanoparticles in polymeric/rubber asphalt binders, in certain percentages, can amplify the elastic capacity of the matrix [52].

**Figure 6:**
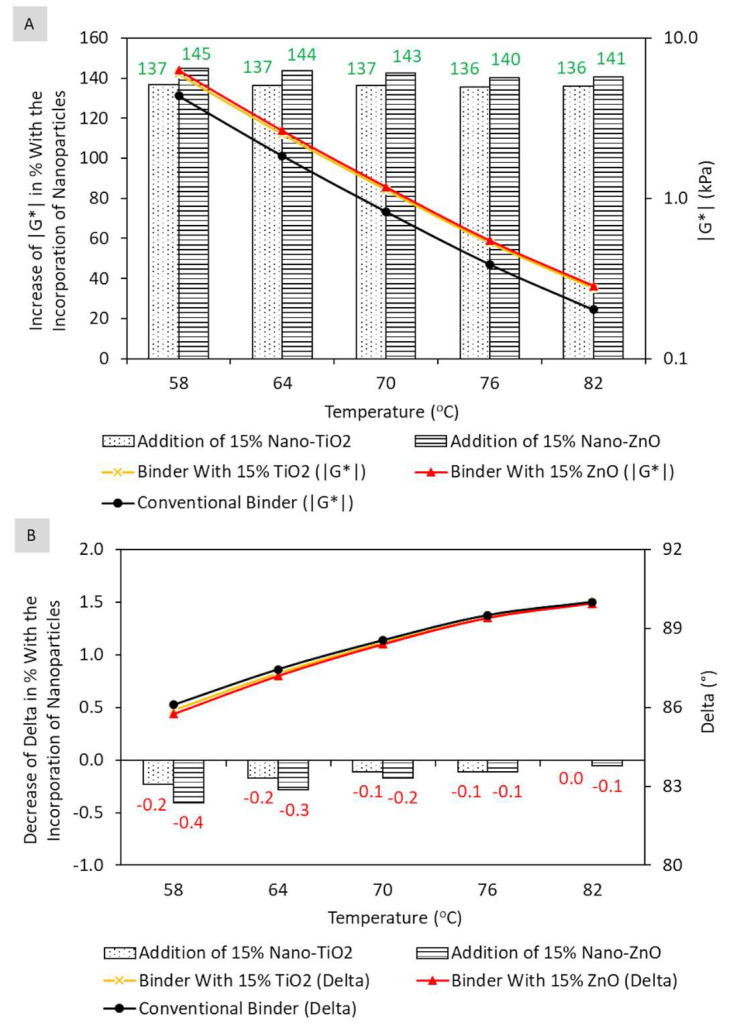
Influence of the incorporation of 15% of nanoparticles on the dynamic shear modulus (|G*|) (**A**) and phase angle (δ) (**B**) of the asphalt matrix aged in the short term at high temperatures.

Finally, with regard to the comparison between TiO_2_ and ZnO, two behavior phases stand out: one with incorporations smaller than 7%, where nanoparticles present similar performance, and another, with additions greater than 7%, where ZnO makes the matrix stiffer when compared to TiO_2_. The beginning of a plateau from 12% incorporation is also observed, with peak at 15%, regardless of type of nanoparticle. The performance peak represents the maximum values of rheological parameter |G*|/sin δ, indicative of the contribution of the binder to permanent deformation, guaranteeing these composites (with 15% TiO_2_ and 15% ZnO) less susceptibility to permanent deformation when compared to the other matrices. In this context, Figure 6 shows the impact of TiO_2_ and ZnO incorporations in the peak region (15% of nanoparticles) in terms of |G*| and δ.

As shown in Figure 6A, the incorporation of 15% of nanoparticles in the asphalt binder, considering all temperatures, causes an average increase in the dynamic shear modulus of 136.3 ± 0.4% when using nano-TiO_2_ and 142.4 ± 1.9% when using nano-ZnO. Regarding the phase angle, it is possible to verify (Figure 6B) average delta reduction of 0.13 ± 0.09% with nano-TiO_2_ and 0.21 ± 0.14% with nano-ZnO, considering all temperatures. Thus, as the change in the phase angle with nanomodification is negligible, the parameter |G*|/sin δ increases practically in the same proportion as the dynamic shear modulus, that is, by 136.3 ± 0.5% and 142.6 ± 2.0%, when incorporating 15% nano-TiO_2_ and 15% nano-ZnO, respectively.

Mathematical equations used to predict the parameter |G*|/sin δ of the aged binder (RTFOT) as a function of temperature and the percentage of nano incorporation are presented by Equations (1) and (2). These equations allow to predict the classification of any conventional binder (PG 58-XX) in relation to the High Temperature Performance Grade (PGH) when TiO_2_ or ZnO nanoparticles are incorporated.

(1) $$\frac{\left|G*\right|}{\sin\delta }{\;\{\%\mathrm{TiO}}_{2},\;T\}=\frac{\left({77.14\;\times \;10}^{13}{\;\times \;\%\mathrm{TiO}}_{2}\right)+({23.82\;\times \;10}^{15})}{T^{\left({0.0014\;\times \;\%\mathrm{TiO}}_{2}\right)+8.9219}}$$

(2) $$\frac{\left|G*\right|}{\sin\delta }\;\{\%\mathrm{ZnO},\;T\}=\frac{\left({18.56\;\times \;10}^{14}\;\times \;\%\mathrm{ZnO}\right)+({23.82\;\times \;10}^{15})}{T^{\left(0.0066\;\times \;\%\mathrm{ZnO}\right)+8.9219}}$$

where:|G*|/sin δ = is the rheological parameter (kPa) T = is the temperature (°C).

**Figure 7:**
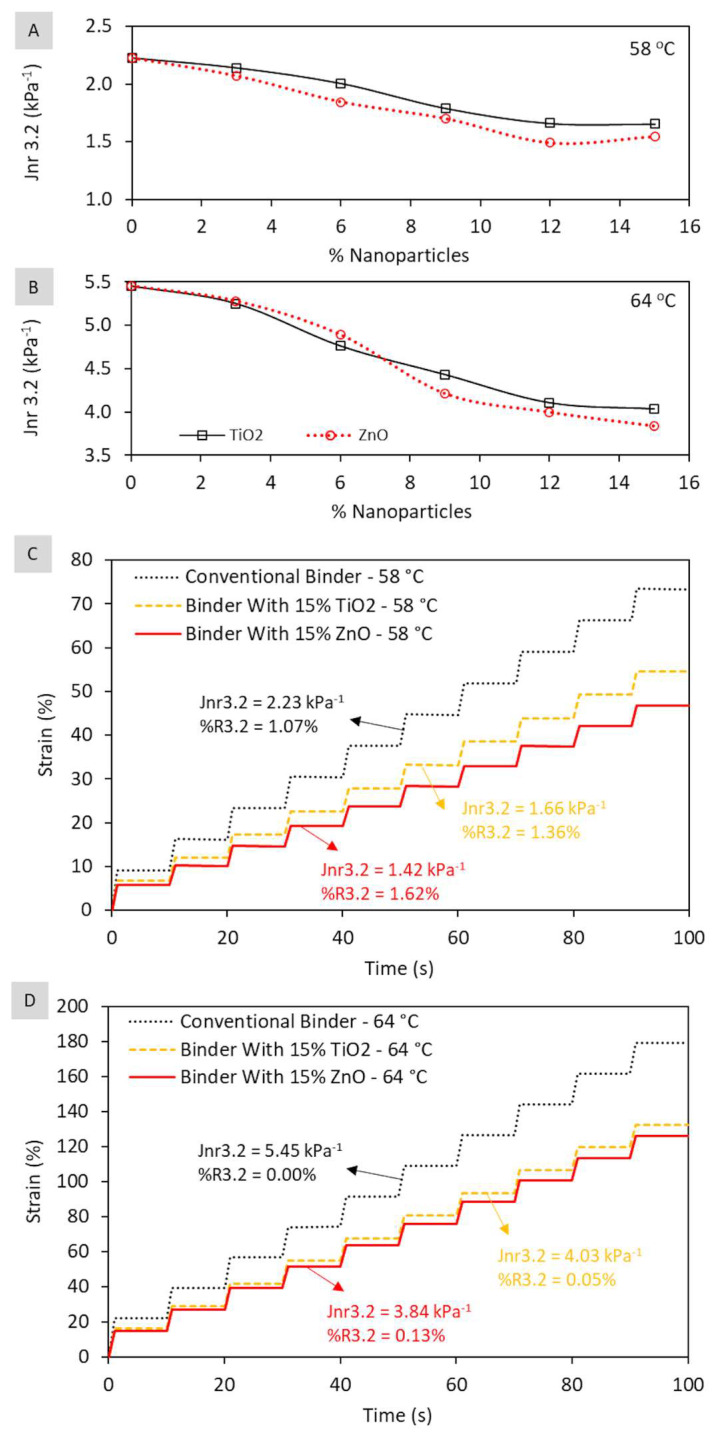
Behavior of asphalt matrices (after RTFOT) in terms of creep and recovery during the MSCR test at stress of 3.2 kPa: non-recoverable creep compliance as a function of the percentage of nanoparticle at temperature of 58 °C (**A**) and at temperature of 64 °C (**B**) and comparison of creep and recovery curves of the conventional binder in relation to composites with 15% TiO_2_ and 15% ZnO at temperature of 58 °C (**C**) and at temperature of 64 °C (**D**).

Additionally, Figure 7 presents the results regarding the Multiple Stress Creep and Recovery (MSCR) test at stress of 3.2 kPa (average of 10 cycles), performed immediately after 20 cycles of creep and recovery at stress of 0.1 kPa. Each value is characteristic of the average of two short-term aged samples.

According to Figure 7, it is possible to verify for both investigated temperatures (58 °C and 64 °C) that the conventional binder presents the worst behavior in relation to the non-recoverable creep compliance (Jnr_3.2_). At a temperature of 58 °C, Jnr_3.2_ was 2.23 kPa^−1^ for the conventional binder, 1.66 kPa^−1^ for binder with nano-TiO_2_ (15%), and 1.42 kPa^−1^ for binder with nano-ZnO (15%). As the temperature increased to 64 °C, Jnr_3.2_ increased for all matrices, resulting from the thermal susceptibility of the asphalt. However, the performance hierarchy remained, where Jnr_3.2_ was 5.45 kPa^−1^ for the conventional binder, 4.03 kPa^−1^ for binder with nano-TiO_2_ (15%), and 3.84 kPa^−1^ for binder with nano-ZnO (15%). Considering both temperatures, the addition of nanoparticles caused an average reduction of the parameter in relation to the conventional binder of 25.8 ± 0.3% when adding nano-TiO^2^ and 33.0 ± 4.7% when adding nano-ZnO. As for the recovery rate (%R_3.2_), all matrices showed values below 2%, characterizing low elasticity, confirming that these nanoparticles are not able to provide elasticity to the matrix.

The better performance of composites in the test is due to the greater stiffness of these matrices as a result of the nanometric reinforcement. This finding is confirmed by analyzing the final strain value (ε_1_) of binders after applying a stress of 3.2 kPa (constant) for 1 s. Considering the 10 loading cycles (3.2 kPa), the mean values for each matrix were the following: (a) at 58 °C: ε_1_ = 7.20 ± 0.05% (conventional binder); ε_1_ = 5.38 ± 0.05% (composite with TiO_2_); ε_1_ = 4.61 ± 0.06% (composite with ZnO); (b) at 64 °C: ε_1_ = 17.41 ± 0.13% (conventional binder); ε_1_ = 12.91 ± 0.14% (composite with TiO_2_); and ε_1_ = 12.29 ± 0.25% (composite with ZnO). In summary, considering both temperatures, matrices nanomodified with TiO_2_ and ZnO present deformation 25.6 ± 0.4% and 32.7 ± 3.4% less than the conventional matrix. Thus, the results obtained in the MSCR test corroborate the behavior observed in matrices regarding the dynamic shear modulus (|G*|) and phase angle (delta—δ). It could be concluded that composites are less susceptible to permanent deformation than the conventional binder. The performance gain of composites is related to the nature of nanoparticles, and both are small in size, have high specific surface area, crystalline, with high mechanical strength and structural stability, allowing the reinforcement of the matrix nanostructure.

Equations (3) and (4) show the non-recoverable creep compliance (Jnr_3.2_) prediction equations of the aged binder (RTFOT) as a function of temperature and percentage of nanoparticles (TiO_2_ or ZnO) added to the matrix. The equations allow to predict the classification of any conventional binder (PG 58-XX) with TiO_2_ or ZnO nanoparticles in relation to the traffic level proposed by the AASHTO M 332 standard [48].

(3) $$J_{\mathrm{nr}3.2}\;\left\{{\%\mathrm{TiO}}_{2},\;T\right\}=\left[\left(-{9.33\;\times \;10}^{-19}{\;\times \;\%\mathrm{TiO}}_{2}\right)+\left({1.97\;\times \;10}^{-16}\right)\right]T^{(-{0.0033\;\times \;\%\mathrm{TiO}}_{2})+9.1}$$

(4) $$J_{\mathrm{nr}3.2}\;\{\%\mathrm{ZnO},\;T\}=\left[\left(-{7.81\;\times \;10}^{-18}\;\times \;\%\mathrm{ZnO}\right)+\left({1.97\;\times \;10}^{-16}\right)\right]T^{(0.0093\;\times \;\%\mathrm{ZnO})+9.1}$$

where: J_nr3.2_ = non-recoverable creep compliance at 3.2 kPa creep stress (kPa^−1^) and T = is the temperature (°C).

Regarding the binder Aging Index (AI) calculated based on the rheological parameter |G*|/sin δ in the virgin and aged state (RTFOT), considering all test temperatures (58 °C to 82 °C), a reduction in the index was observed with the addition of nanoparticles. The average index was 2.00 ± 0.10 for the conventional matrix, 1.79 ± 0.07 for composite with 15% TiO_2_, and 1.67 ± 0.10 for composite with 15% ZnO. In percentage terms, the reduction in the index is 10.7 ± 0.8% and 16.6 ± 0.7% when TiO_2_ and ZnO are incorporated, respectively. It could be concluded that composites are more resistant to aging. The greater resistance is a function of the special characteristics of nanoparticles: the particle size allows to fill the matrix, blocking oxidation and volatilization; the high specific surface area and surface energy promote adsorption inside the matrix, reducing the exudation of oily compounds that adhere to the surface of particles.

Equations (5) and (6) show a model for TiO_2_ and another for ZnO for predicting the influence of nanoparticles on the Aging Index (AI) for any conventional binder (PG 58-XX) as a function of temperature and percentage of nano incorporation.

(5) $${\mathrm{AI}\;\{\%\mathrm{TiO}}_{2},\;T\}=\frac{\left(-{0.1985\;\times \;\%\mathrm{TiO}}_{2}\right)+9.3491}{T^{\left(-{0.0043\;\times \;\%\mathrm{TiO}}_{2}\right)+0.364}}$$

(6) $$\mathrm{AI}\;\{\%\mathrm{ZnO},\;T\}=\frac{\left(0.0255\;\times \;\%\mathrm{ZnO}\right)+9.3491}{T^{\left(0.0035\;\times \;\%\mathrm{ZnO}\right)+0.364}}$$

where: AI = Aging Index; T = is the temperature (°C).

### 3.2. Performance of Composites Regarding Fatigue Damage Tolerance

**Figure 8:**
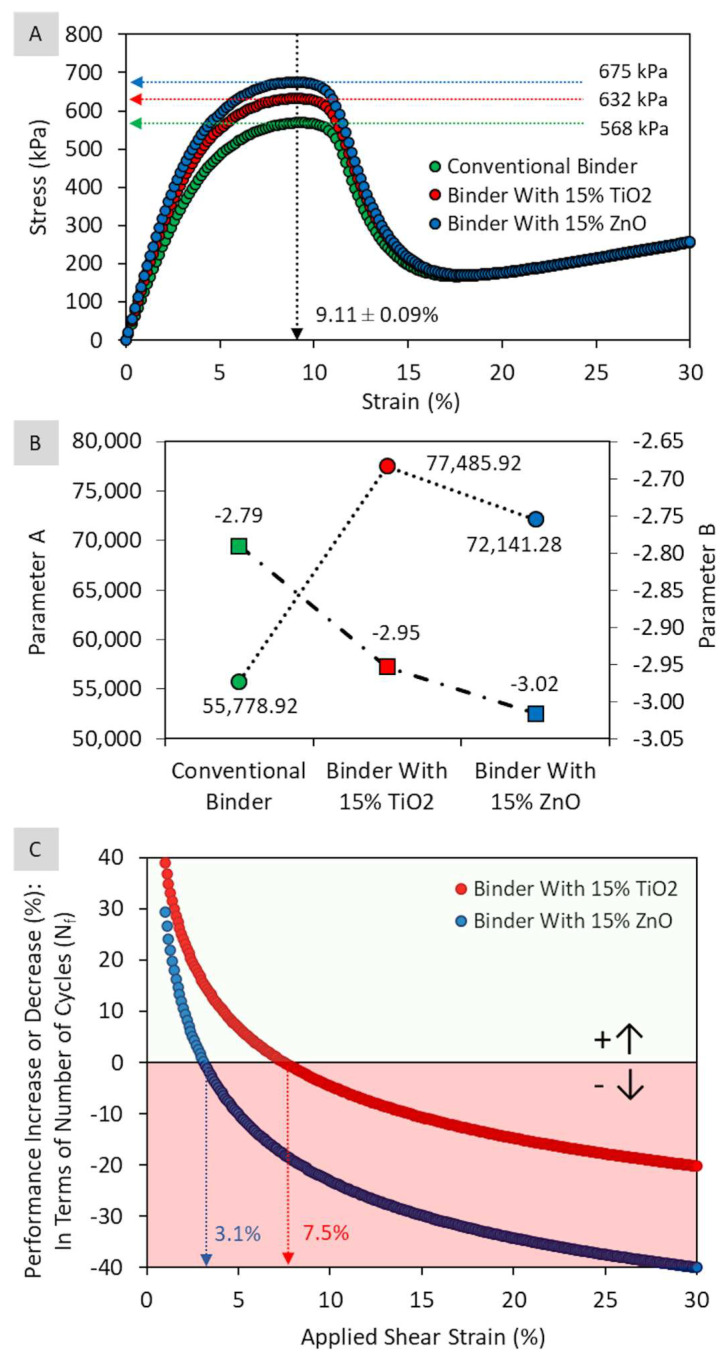
Results of the LAS test (20 °C): (**A**) stress-strain curve, (**B**) parameters A and B of the fatigue model, and (**C**) effect of the type of nanoparticle on the damage tolerance of the asphalt matrix (increase or decrease in performance) as a function of the deformation amplitude.

The effect of adding the two types of nanoparticles on the matrix behavior in terms of damage tolerance was verified by the LAS test (20 °C). Figure 8 presents the results obtained in terms of the stress–strain curve, parameters A and B of the fatigue model (Nf = A(γ_max_)^B^), and percentage increase or decrease in damage tolerance (Nf) as a function of the deformation amplitude and type of nanoparticle.

According to the stress–strain curve shown in Figure 8A, the addition of nanoparticles at 15% content increases the matrix stiffness at intermediate temperatures, as the slope of the curve in the elastic region is more pronounced for composites with ZnO and TiO_2_, respectively. This statement is corroborated by the maximum stress peak, with higher values for ZnO (675 kPa, 18.7% higher) and TiO_2_ composites (632 kPa, 11.1% higher) compared to the conventional binder (568 kPa) in the same deformation amplitude (9.11 ± 0.09%). Comparing nanoparticles, the sample with 15% zinc oxide has a maximum stress 6.8% higher than sample with 15% titanium dioxide.

As for the fatigue model (Figure 8B), it appears that the incorporation of nanoparticles increases parameter A, where binders with nano-TiO_2_ and nano-ZnO showed, respectively, an average increase of 38.9% (77,485.92) and 29.3% (72,141.28) in relation to the reference (55,778.92). For parameter B, there is an increase in value (in modulus) when nanoparticles are added to the asphalt matrix. The sample with zinc oxide had a result 8.1% (|−3.02|) higher than the reference (|−2.79|), whereas the composite with titanium dioxide showed a value 5.8% (|−2.95|) higher. Finally, it appears that the presence of both nanoparticles increases the matrix sensitivity to the level of deformation, with greater intensity for nano-ZnO.

In this perspective, Figure 8C highlights the impact of incorporations on the fatigue damage tolerance as a function of the deformation amplitude. At deformation amplitudes greater than 3.1% for binder with ZnO and 7.5% for binder with TiO_2_, a reduction in the number of cycles for failure (Nf) is observed when compared to the reference, where the greater the deformation amplitude, the smaller the performance of composites. Evaluating the type of nanoparticle, it was observed that the matrix modified with TiO_2_ presents better fatigue damage tolerance than the matrix with ZnO, where, considering all deformation amplitudes, the number of cycles until failure is on average 25.9 ± 6.0% higher.

It could be concluded that the addition of nanoparticles in the asphalt matrix is beneficial in terms of fatigue damage tolerance at small deformation amplitudes, impairing the resulting behavior at higher deformation amplitudes. In this case, for amplitudes of 15%, pointed out by Chen, Zhang, and Bahia [50] and Chen and Bahia [51] as a region with stronger correlation with the fatigue life of asphalt mixtures, there is strong indication of a negative effect of the incorporation of these two nanoparticles. Composites would present performance loss in relation to the conventional binder in the order of 10.6% and 28.8%, when incorporating nano-TiO_2_ and nano-ZnO in the asphalt matrix, respectively. However, it is emphasized that there are no studies in literature on the effective prediction of parameters obtained in the LAS test regarding nanomodified mixtures.

Equations (7) and (8) show performance prediction equations regarding fatigue for conventional asphalt binders (PG 58-XX) modified with nano-TiO_2_ and nano-ZnO. The equations provide the number of cycles (Nf) as a function of the maximum deformation amplitude (γ_max_) and nanoparticle content (%TiO_2_ or %ZnO).

(7) $$N_{f}\;\left\{{\%\mathrm{TiO}}_{2}{,\;\gamma }_{\max}\right\}=[{1378.68(\%\mathrm{TiO}}_{2})+55,778.92]{\left(\gamma_{\max}\right)}^{-{0.0106(\%\mathrm{TiO}}_{2})\;-\;2.79}$$

(8) $$N_{f}\;\left\{{\%\mathrm{ZnO},\;\gamma }_{\max}\right\}=[1161.01(\%\mathrm{ZnO})+55,778.92]{\left(\gamma_{\max}\right)}^{-0.0147(\%\mathrm{ZnO})\;-\;2.79}$$

where: N_f_ = number of cycles until failure; γ_max_ = Strain amplitude.

## 4. Conclusions

This study evaluated the rheological behavior of conventional asphalt binders modified with TiO_2_ and ZnO nanoparticles and proposed mathematical equations for performance prediction. According to the results obtained, it could be concluded that:

- The increase in the nano-TiO_2_ and nano-ZnO content in the asphalt matrix results in the improvement of rheological parameters related to performance at high temperatures, specifically parameter |G*|/sin δ and the non-recoverable creep compliance at a stress of 3.2 kPa. This indicates a potential gain in resistance to permanent deformation of the asphalt material;
- With the incorporation of nanoparticles, the porosity of the conventional asphalt binder was filled due to the reduced dimensions of nanoparticles (TiO_2_ and ZnO), which increased impermeability and reduced the effects of oxidation and volatilization, reflecting in the reduction of the Aging Index values;
- In terms of fatigue resistance, when compared to the pure asphalt binder, both composites show increase in the number of cycles until failure at low deformation amplitudes (up to 7.5% for binders with nano-TiO_2_ and up to 3.1% for binders with nano-ZnO). On the other hand, at high deformation amplitudes, the opposite was observed. This behavior is intensified as the nanoparticle content increases. This points to an increase in sensitivity to the deformation level;
- In the comparison between composites, the binder modified with nano-TiO_2_ presents a higher number of cycles during the assessment of the fatigue damage tolerance, whereas the binder with nano-ZnO indicates better mechanical and rheological response at high temperatures; and
- It was possible to define mathematical performance equations for each rheological parameter, providing a perspective for predicting the behavior of conventional asphalt binders (PG 58-XX) when modified with nano-TiO_2_ or nano-ZnO.

## Figures and Tables

**Table 1:** Asphalt binder properties.

Properties	Results
High Temperature Performance Grade [26]	PG 58-XX
Penetration [27]	60 × 10^−1^ mm
Softening point [28]	49.5 °C
Apparent viscosity at 135 °C (20 rpm) [29]	0.326 Pa·s
Apparent viscosity at 150 °C (50 rpm) [29]	0.178 Pa·s
Apparent viscosity at 177 °C (100 rpm) [29]	0.064 Pa·s
Flash point [30]	280 °C
Relative density at 20 °C [31]	1.012
Penetration Index (Pfeiffer e Van Doormaal) [32]	−0.9

## Data Availability

The data presented in this study are available on request from the corresponding author.
